# A Review on the Analysis of Thermal and Thermodynamic Aspects of Grain Refinement of Aluminum-Silicon-Based Alloys

**DOI:** 10.3390/ma16165639

**Published:** 2023-08-15

**Authors:** Ehab Samuel, Agnes M. Samuel, Victor Songmene, Fawzy H. Samuel

**Affiliations:** 1 Département des Sciences Appliquées, Université du Québec à Chicoutimi, Chicoutimi, QC G7H 2B1, Canada; ehabfhsamuel@gmail.com (E.S.); agnesmsamuel@gmail.com (A.M.S.); 2 Department of Mechanical Engineering, École de Technologie Supérieure (ÉTS), Montréal, QC H3A 1K3, Canada; victor.songmene@etsmtl.ca

**Keywords:** grain refining, Al_3_Ti, AlB_2_, undercooling, Ti-Si interaction, microstructure

## Abstract

The present analysis addresses the solidification and thermodynamic parameters involved during the solidification of aluminum (Al)-based alloys as presented in the literature using different systems viz., binary aluminum-boron (Al-B) and aluminum-titanium (Al-Ti) systems, ternary aluminum-titanium-boron (Al-Ti-B) and aluminum-titanium-carbon (Al-Ti-C) systems, as well as taking into consideration the silicon-titanium-aluminide (Si-TiAl_3_) interaction in Al-based alloys containing Si. The analysis is supported by recent metallographic evidence obtained by the authors on A356.2 alloys. The sections on thermodynamic aspects cover the different models proposed concerning nucleation and growth on a newly formed Al grain. The value of the recalescence parameter reduces gradually with the increase in the Ti added. At a level of 0.20 wt%, this parameter becomes zero. If the concentration of grain refiner exceeds a certain amount, the grain size becomes minimal. Another parameter to be considered is the interaction between the grain refiner and traces of other metals in the base alloy. For example, Al-4%B can react with traces of Ti that may exist in the base alloy, leading to the reaction between boron and titanium to form titanium diboride (TiB_2_). Grain refinement is achieved primarily with TiB_2_ rather than aluminum diboride (AlB_2_), or both, depending on the Ti content in the given alloy.

## 1. Introduction

Although in tonnage, the production of aluminum (Al) represents only a little more than 2% of that of steel, this metal (and the alloys which derive from it) arrives in second position with regard to the production and the use of metallic materials. Aluminum owes this place to a set of properties which, in many circumstances, make it an irreplaceable material. These include the density, the electrical conductivity, and the mechanical and shaping properties [1]

However, for various reasons, as with other metals, Al is rarely used in its pure state. Various elements are added to Al to modify or improve the mechanical properties. One of the main elements added to Al is silicon (Si). Moreover, 85 to 90% of the parts produced in aluminum are made of aluminum-silicon (Al-Si) alloys. They offer excellent castability and can be machined and welded. In addition to Si, there is also titanium (Ti), which can be alloyed with aluminum. Its presence has consequences on solidification, microstructure, and mechanical properties. Like all metals, the macrostructure of aluminum is made up of multiple crystals and grains that are formed during solidification. Their final size results from phenomena that take place only at the very beginning of solidification. Increasing the solidification rate or applying vibration also reduces the grain size. Another method consists in adding particles to an alloy which facilitate the nucleation and consequently the refining of the aluminum grains. These particles are added using different master alloys. The solidification of any metal or alloy can be studied by thermal analysis. The latter consists of analyzing the temperature data as a function of time (T(t)), which is collected during solidification [2,3,4].

Grain refining in aluminum alloys aims to increase the number of crystallization sites of the proeutectic phase (α-Al phase) and avoid columnar growth. In order to have a fine scale grain size, the most widely practiced method is to present effective nuclei in the liquid metal using the Al-Ti-B grain refiners, which usually contain active seeds like TiAl_3_, TiB_2_, AlB_2_, or (Al,Ti)B_2_. Thermodynamic studies suggest that these latter particles convert to TiB_2_, so that the titanium would diffuse into the (Al,Ti)B_2_ particles while the aluminum diffuses out, resulting in the formation of TiB_2_ [3,4,5].

One of the main problems in obtaining grain refinement is the contact time between the nucleation sites and the liquid metal, which must not exceed a critical threshold. If this contact time is too short, a finer grain size cannot be achieved. On the other hand, if this time is too long, the efficiency of the grain refiner will be lowered. The agglomeration and arrangement of TiAl_3_ and TiB_2_ particles is the main reason for such erasure. Stirring may help overcome erasing issues. If the size of the grains depends strongly on various factors—namely, size, quantity and morphology of the nucleating substrates, nature of the master alloy, holding time before casting, overheating temperature, cooling rate, type of mold used, and silicon content—then it also plays a major role in determining grain size. Indeed, when silicon is added, it reduces the grain size by what are called constitutional or restriction growth effects. However, above around 3 wt% silicon, the increase in grain size occurs. It has been suggested that the TiB_2_ interface constitutes an energetically favorable location for silicon atoms, compared to the boundaries of the subgrain/grain matrix. Another factor which could be of importance is that the solubility of titanium in solid aluminum decreases with the presence of silicon [6,7].

While the Al-Si alloy system is widely used in industry, constituting approximately 85–90% of aluminum parts produced, the eutectic silicon in untreated alloys is often very coarse, leading to poor mechanical properties, especially ductility. These properties are strongly influenced by the morphology of the eutectic silicon. The latter changes from its original raw structure of platelets to a less harmful and finer fibrous structure, termed eutectic modification, which leads to a significant improvement in the mechanical properties of the products. The modification of the eutectic silicon is usually accomplished by adding certain modifying agents such as strontium (Sr). However, over-modification can lead to the formation of porosities and the return of the silicon to its original shape, again weakening the characteristics of the alloy [8].

The addition of strontium in Al-Si alloys leads to a considerable increase in the amount of the α-Al dendritic phase and changes the shape of the dendrites. In the presence of a grain refiner like Al-Ti-B, the reduction in modification is considerable since the Sr-Ti interaction alternately changes the volume fraction of the dendritic α-Al phase and the morphology of the silicon phase [9,10].

Using cleaner Ti-5B master alloy with a higher number of TiAl_3_ and TiB_2_ particles improves its grain refinement efficiency and transmits fewer impurities in produced parts. Producing a cleaner master alloy would be beneficial from economic and environmental aspects by increasing its value and the service time of the produced parts, as well as simplifying the recycling process at the end of the life-cycle of these parts [11].

## 2. Objectives

The purpose of this research project is to study grain refinement by Ti/B as well as the different effects of the presence or increase in the concentration of these elements in the A356.2 alloy. The main objective is to examine the effects of increasing the concentration of the added Ti, B, or B + Ti on:

1. Reducing the grain size compared to other elements.
2. Solidification by thermal analysis, including phenomena and parameters that take place during solidification at slow rate (~1 °C/s).
3. The alloy microstructure and whether there is an effect on the alloy mechanical behavior.
4. Intermetallic formation. Once the grain refiner is present in the alloy, the intermetallics associated with the additions become an integral part of the microstructure. Therefore, it is then possible to study them under various experimental conditions. In addition, it is possible to study the interaction of these intermetallics with the different components of the microstructure.
5. The interaction between the Sr modifier and the grain refiner (Al-10Ti, Al-5Ti-1B, Al-4B), the interaction between silicon and titanium, and their competing influence on the morphology of the eutectic silicon, on the size of the grains and on the shape of the dendritic α-Al phase.

### 2.1. Thermal Analysis Method

**Figure 1:**
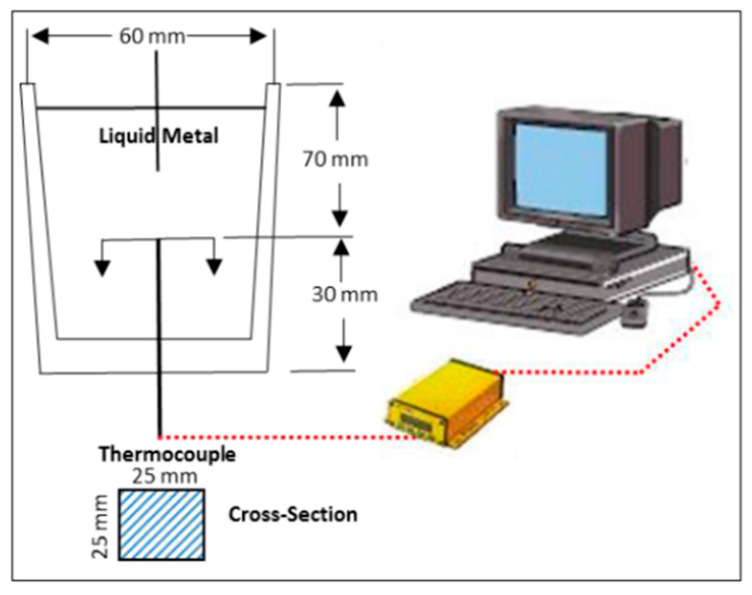
Schematic drawing showing the graphite mold used for thermal analysis.

Master alloys containing titanium were added to the A356 alloy, so that the added melt would contain Ti values of: 0, 0.1, 0.2, 0.3, 0.4, 0.6, and 1.0% (all values are in weight %) in order to comply with the usual addition standards and to meet industrial needs. After the addition of the grain refiner, the molten metal was stirred for about 30, before casting the liquid metal in a graphite mold preheated to 600 °C—Figure 1. The data relating to the thermal analysis were measured using type K thermocouples, 0.3 mm in diameter. During cooling, data were recorded using a computer capable of high-speed data acquisition at five measurements per second. For the Al-4%B alloy, the additions were made in a sequence ranging from 0 ppm to 1000 ppm B. The contact time between the liquid metal and the AlB2 boron nucleation sites before casting was d ½ hour with a suitable stirring period. Given the quantity of the liquid bath (800 g), this stirring period did not exceed 10 min. Metallographic samples were sectioned from these castings, polished, and etched in a solution made of (66% HN03, 33% HCl, 1% HF). Thereafter, the samples were examined employing a scanning electron microscope equipped with an energy dispersive X-ray (EDS) system. Table 1 lists the chemical composition of the A356.2 used in the present investigation.

### 2.2. Thermal Concept

As described in the previous Section 2.2, thermal analysis makes it possible to study different phenomena and parameters that occur during solidification. An alloy is made up of several elements, in general. Not only is the presence of these elements important, but also the manner in which they are distributed. The study of the microstructure therefore consists in analyzing the different components of the microstructure (α-Al matrix, intermetallics, etc.), formed according to the experimental parameters. Moreover, the addition of particles for grain refinement can change the composition of the alloy and, therefore, the microstructure. The eutectic reaction in a binary system may be described as follows:

Liquid → α + β (1)

An alloy in the liquid state of composition C_E_ (the eutectic concentration) will solidify at a constant temperature into two distinct solid phases, α and β, of composition Cα and Cβ, respectively. There is also the monotectic reaction:

Liquid 1 → γ + Liquid 2(2)

After this reaction, a solid phase (γ) is in equilibrium with the molten alloy (Liquid). In addition, some systems include a peritectic reaction. This reaction involves the transformation of a solid phase and a liquid phase into a new single solid phase. This reaction can be written as:

Liquid + δ → ε(3)

This reaction occurs at a constant temperature. At the peritectic point, three phases are in equilibrium.

## 3. Al-B and Al-Ti Systems

For this system, during solidification, the following eutectic reaction is observed [1,2]:

Liquid → α-Al + AlB_2_ (4)

**Figure 2:**
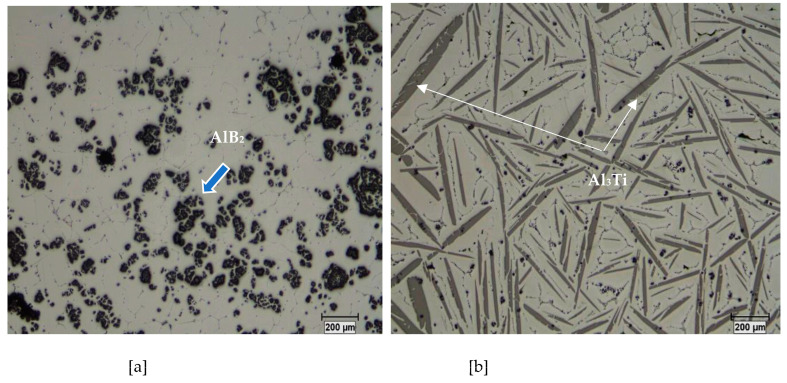
Microstructures of: (**a**) Al-4 wt.%B, (**b**) Al-10 wt.%Ti master alloys.

**Figure 3:**
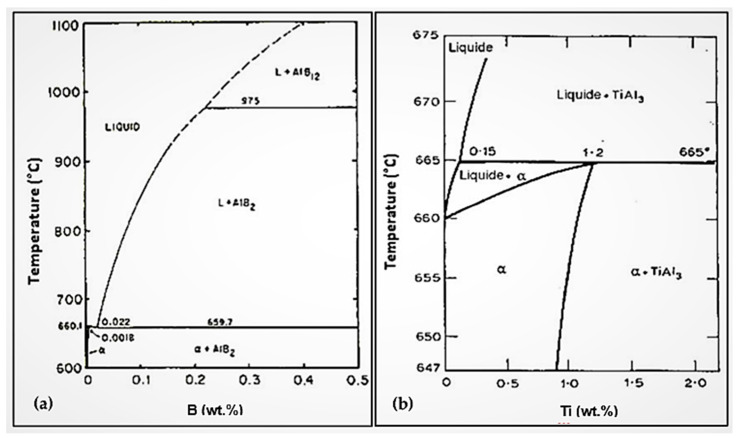
(**a**) Low boron portion of the Al-B equilibrium diagram; (**b**) Low boron portion of the Al-Ti equilibrium diagram.

Depending on the temperature and the Ti concentration (Figure 2), there occurs formation of TiAl_3_ intermetallics following the monotectic reaction as shown in Figure 3a,b:

Liquid 1 → Liquid 2 + TiAl_3_ (5)

If the temperature decreases further, there occurs the following peritectic reaction [3,4,5,6]:

Liquid 2 + TiAl_3_ → α-Al (6)

**Figure 4:**
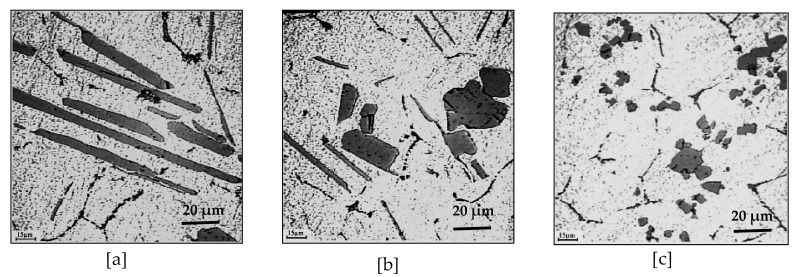
Microstructures of Al-Ti master alloys: (**a**) TiAl_3_ -acicular particles; (**b**) TiAl_3_-mixed shapes (acicular and blocky particles); (**c**) TiAl_3_ -blocky particles (data from [13]).

**Figure 5:**
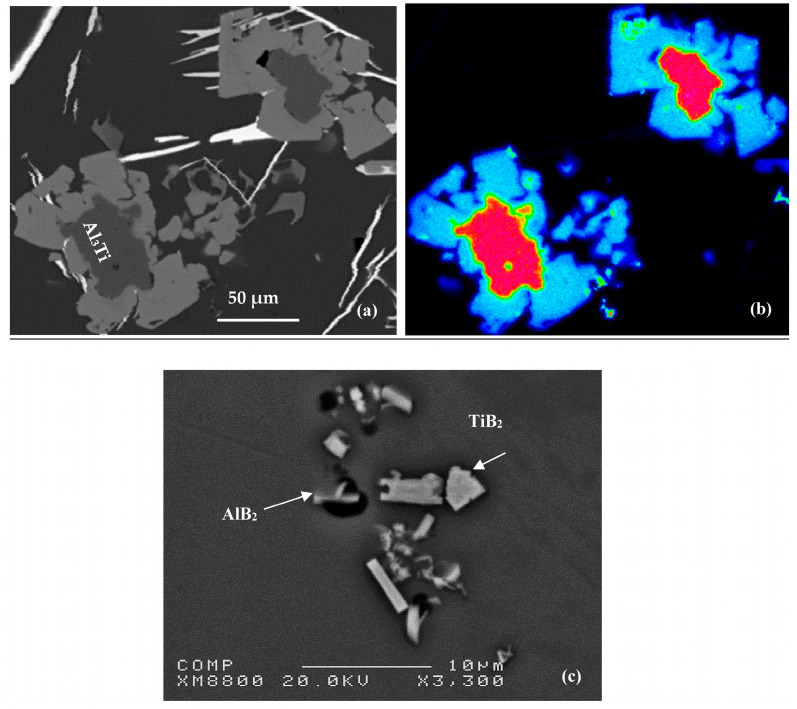
Precipitation of other phases or α-Al on the Al_3_Ti particles in Al-Si alloys: (**a**) backscattered electron image, (**b**) X-ray image of Ti in Al_3_Ti, (**c**) simultanious presence of Ti and B.

Li et al. [12] investigated the effect of various microstructures on grain refinement by Al-Ti master alloys synthesized at high temperature by mixing aluminum with titanium. The results of their work show that the variation in experimental parameters, such as the stoichiometric ratio of the initial powders, the particle sizes of the powders, the use of fluxes, etc., led to the formation of various structures of the master alloys, in particular sizes, morphologies, and quantities of Al_3_Ti particles. The intermetallic particles showed a needle-like (acicular) morphology in the lack of aluminum powder in the initial mixture, which was related to the higher reaction temperature (see Figure 4a). The same particles had needle-like and blocky appearances at the same time (mixed morphology), which resulted from the lower reaction temperature (see Figure 4b). Block-like Al_3_Ti crystals were formed at a lower reaction temperature when excess aluminum powder was added to the initial mixture (see Figure 4c). Figure 5 displays the decomposition of Al-Ti and Al-B master alloys after addition to the A356 alloy.

## 4. Al-Ti-B System

**Figure 6:**
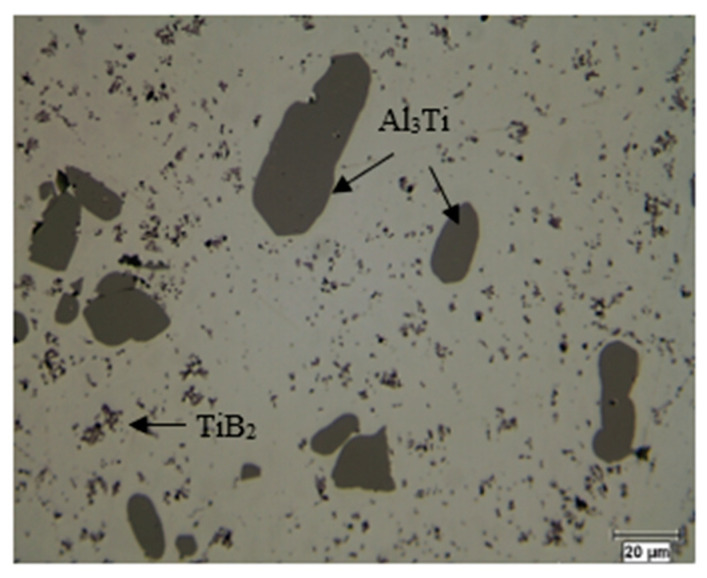
Optical micrograph revealing the microstructure of the Al-5%Ti-1%B master alloy.

Figure 6 illustrates the microstructure of the Al-5%Ti-1%B master alloy. The latter contains Al_3_Ti-type particles having a blocky morphology with an average diameter of 50 µm. In the presence of the Al_3_Ti phase, a second phase of the TiB_2_ type coexists in the microstructure, and this is proven by X-ray diffraction analysis. Both phases are dispersed in the aluminum matrix. For this system, several phenomena are possible. Depending on the value of certain parameters, the phenomena observed during solidification will be different. These parameters are the titanium concentration ([Ti]), the boron concentration ([B]), and the stoichiometric ratio Ti:B [7,8,9,10]. If the concentrations are high enough, there is first the formation of TiB_2_ particles. Moreover, if the stoichiometric ratio is high, there is a surplus of Ti and the solidification of the alloy is accompanied by the monotectic and peritectic reactions associated with titanium [11,12,14,15,16,17,18]. Conversely, if the Ti:B ratio is low (titanium deficiency), there is formation of a metastable phase (Al,Ti)B_2_ [10,19,20,21]. Furthermore, if the ratio is even lower, there is formation of AlB_2_ during solidification.

**Figure 7:**
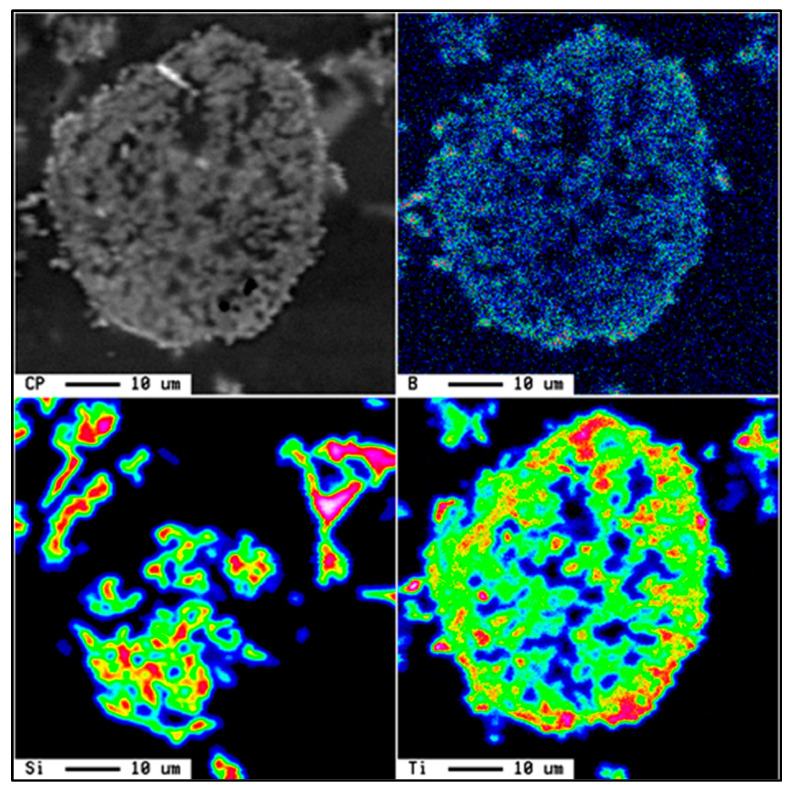
An X-ray map showing the distribution of Ti, Si, and B.

When an Al-Ti-B-type master alloy is added to an alloy such as A356 (~7%Si), several intermetallic phases are formed. Among these, mention is made of the intermetallics of the (Al,Si)_3_Ti and Ti_6_Si_2_B types. Ramos et al. [22] carried out detailed research on the ternary phase, Ti_6_Si_2_B, which belongs to the Ti-Si-B system. Based on X-ray diffraction analysis, this phase is characterized by a hexagonal crystalline structure with lattice parameters a = 0.68015 nm and c = 0.33377, and it forms liquid through the following peritectic reaction: L + TiB + Ti_5_Si_3_ 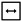 Ti_6_Si_2_B. Figure 7 shows the X-ray mapping of the Al-Ti-Si-B phase.

## 5. Al-Ti-C System

**Figure 8:**
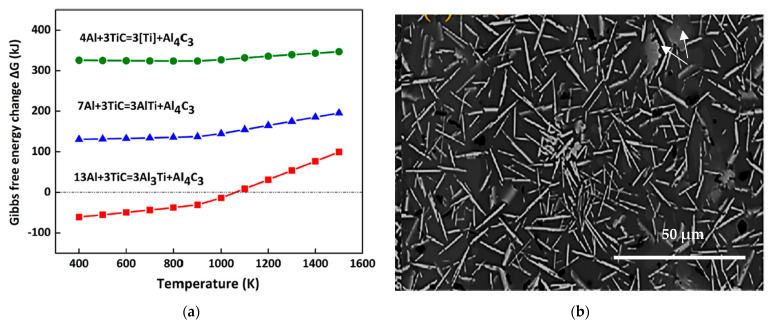
(**a**) Variation of the standard free energy of formation as a function of temperature for TiC and Al_4_C_3_ particles (data from [24]). (**b**) Backscattered electron image of Al-Ti-C master alloy. Arrows point to the platelet-like morphology of TiC particles.

With this system, TiC particles are present in the metal, as displayed in Figure 6. When the temperature is below 1273 K (1000 °C), the TiC particles are unstable and react with liquid aluminum to form aluminum carbides (Al_4_C_3_ and Ti_3_AlC) [23]. It is possible to cancel this negative effect by superheating the molten metal. The temperature must then be increased to more than 1800 K or 1527 °C [23]; see Figure 8a. Thus, if the temperature is relatively low, the following reactions are possible between the TiC particles, Al, and Ti [24]:3TiC + 4Al 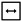 Al_4_C_3_ +3 Ti(7)
9Ti + Al_4_C_3_

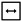
 3Ti_3_AlC + Al(8)

At this point, the presence of fine crystals of Al_4_C_3_ and complex carbides at the Al/TiC interface can be observed. In the case of an Al-7% Si alloy (A356.2), the TiC particles completely decompose to form Al-Ti-Si-type complex intermetallics and aluminum carbides. These Al-Ti-Si intermetallics contain about 1% carbon. Aluminum carbides are normally precipitated at the boundaries of the other phases existing in the alloy. The chemical formula of these carbides varies from Al_4_C_3_ to Ti_3_AlC, while the chemical composition of the Al-Ti-Si intermetallics is as follows: Al-35%Ti-9% Si (mass proportion). Figure 8b presents the microstructure of Al-5%Ti-0.25%C master alloy.

## 6. Si-TiAl_3_ Interaction

**Figure 9:**
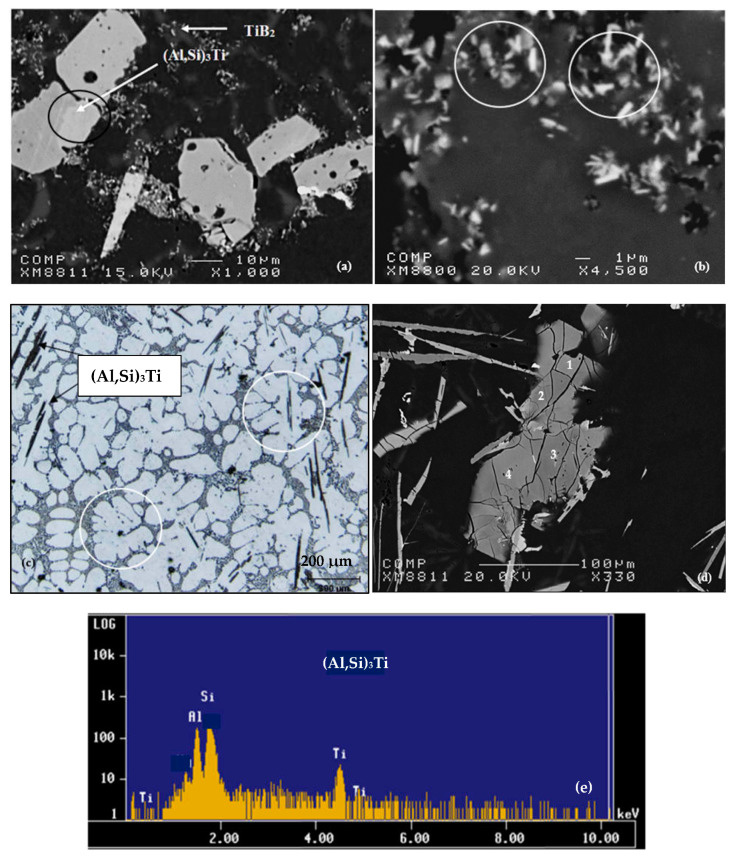
Backscattered electron images depicting: (**a**) agglomeration of (Al,Si)_3_Ti particles; (**b**) precipitation of TiB_2_ particles (white circles), distribution of (Al,Si)_3_Ti platelets in the same alloy; (**c**) optical microstructure revealing the presence of Al_3_Ti platelets within α-Al. (**d**) Same as in (**c**) viewed using electron probe microanalyzer. (**e**) EDS corresponding to (**d**) showing explicit peaks due to Al, Si and Ti.

The addition of TiAl_3_ particles into the A356.2 alloy in the form of the Al-5%Ti-1%B master alloy increases the Ti concentration to 0.4%; see Figure 9a,b. Moreover, if the concentration is high enough, there occurs the formation of TiAl_3_ intermetallics in the alloy during solidification. But the alloy also affects the chemical composition of these intermetallics. Indeed, approximately 9% (mass proportion) of silicon dissolves in the intermetallics of TiAl_3_ [25,26]. The new chemical formula then becomes Ti(Al,Si)_3_ [27]. The morphology of (Al,Si)_3_Ti intermetallics can have a plate shape when the alloy is superheated to 750 °C or a dendrite shape when the superheat temperature is 950 °C. As shown in Figure 9c, these intermetallics, which are the site of the nucleation of the aluminum dendritic phase, are localized to the dendrite zones. The surface fraction of these intermetallics increases with the amount of Al-Ti alloy added to the liquid metal. However, their size does not show a similar trend. This can be directly related to the size of these wafers in the original binary alloy and the conditions of the liquid metal before casting (Figure 9b). It is assumed that these particles (<1 µm in length) are the nucleation sites for the aluminum grains. The density of these particles appears to be related to the amount of Al-Ti binary alloy added to the liquid metal. As can be seen in Figure 9c, the (Al,Si)_3_Ti platelets precipitate within the α-Al (the alloy was grain refined using Al-10%Ti master alloy). Moreover, the dendrites are rounded (white circles). A high magnification electron image of these platelets is shown in Figure 9d, while Figure 9e displays an EDS spectrum corresponding to these platelets using an electron probe microanalyzer, revealing peaks relating to elements of the (Al,Si)_3_Ti phase. Table 2 shows the chemical composition of these platelets.

When solidification starts, the formation of dendrites begins. Initially, these dendrites grow relatively independently, but after a while, they collide. This corresponds to the dendrite coherency point. The metal then has a certain rigidity. In the presence of Ti, the dendrite concordance point occurs at a higher solid fraction [28,29]. In general, in the lit-erature, it is mentioned that overheating increases the size of the grains [30,31,32,33]. In some cases, however, overheating reduces the grain size [34]. In addition, it is often observed that, after degassing, the grain size can increase [31,35]. On the other hand, without degassing, the concentration of impurities rises, which decreases fluidity [36].

**Figure 10:**
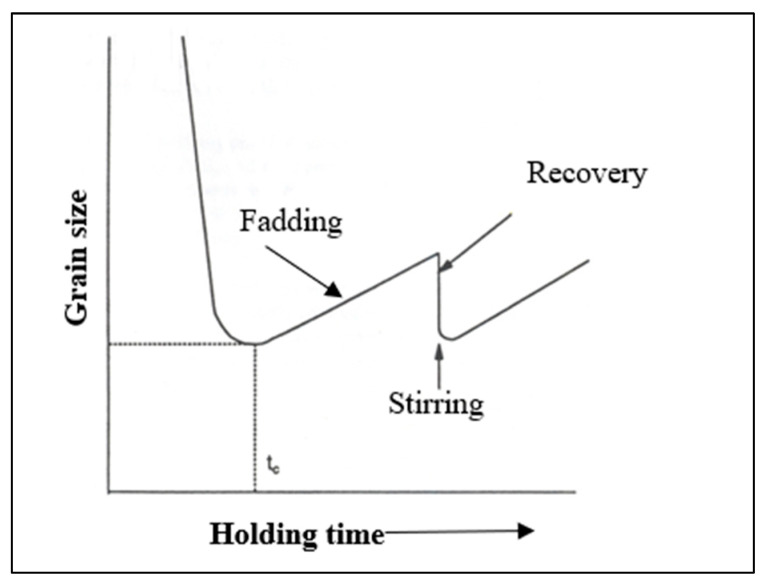
Grain size behavior after addition of grain refiner.

Figure 10 shows a typical grain size variation as a function of holding time after the addition of a grain refiner. The curve is characterized by a rapid decrease in grain size to a minimum value at a holding time, tc, followed by a rise in size at longer times. This refining loss is called fading. The latter can be improved in part by stirring the liquid metal, although the degree of recovery depends on the composition of the molten metal. It is well known that grain refinement can be lost if the metal is held above 750 °C, but this can be regained if more titanium is added and the liquid metal is agitated.

## 7. Nucleation Phenomenon

Alloy A356.2 contains about 0.30–0.45% magnesium (Mg). The presence of this element causes minor modification of the silicon phase particles [37]. For an experimental Al-7% Si alloy, the addition of this element at different concentrations (0.1–0.6%) improves grain refinement [38]. Moreover, the grains are smaller when the Mg concentration is 0.2%. However, whatever the content, degradation (“fading”) occurs after a certain time (≈120 min). Further, the initial improvement at low concentrations of Mg may be related to the restrictive effect of this element (“growth restriction effect”). With regard to mechanical properties, Mg increases the elastic limit and the ultimate limit. For the other elements, the grain size can vary in different ways depending on the concentration. In general, the presence of alloying elements reduces the grain size [38,39]. Note that manganese (Mn) increases grain size [13]. In addition, negative interactions are possible between alloying elements, which can also increase the grain size [40,41,42,43]. Moreover, for iron (Fe), in the presence of Si, its contribution to grain refining is low, even zero or neutral [44]. Some elements may have a particular phenomenology [31,45]. In addition, the ability of elements to refine grains can be related to the following parameters [46]:

Q = m_l_ c_0_ (k − 1) (9)

P = m_l_ c_0_ (k − 1)/k (10)

where m_l_ is the slope of the liquidus, c_0_ the solute concentration in the binary alloy, and k is the partition coefficient. The Q parameter is called the “growth restriction factor”, while the P parameter represents the “undercooling and recalescence parameter” or simply the “undercooling parameter”. The high value of the Q parameter for Ti would explain its efficiency for grain refining [47,48]. On the other hand, the parameter P cannot explain this phenomenon for Ti, unlike for other elements [49]. There is another method for calculating the parameter Q, using the following equation [25,46]:

(11) $$Q=-{\frac{dT}{df_{s}}|}_{Tliq}$$

where *T* is the temperature, *f_s_* the solid fraction, and *T_liq_* is the liquidus temperature. This equation is useful for calculating *Q* for a system whose liquidus and solidus are not linear. It is then possible to generalize the calculation and use of *Q* for multi-component systems. Indeed, for these systems, the global value of *Q* is not necessarily equal to the sum of the values of *Q* of each constituent.

One of the effects of the presence of Ti in an Al alloy is the reduction in grain size. However, the reduction is not continuous and gradual. In the case of pure aluminum, the grain size becomes almost constant when the Ti concentration is around 0.08–0.13% [50]. The grain size even increases slightly if the Ti concentration varies from 0.12 to 0.15%. Grain refining can be described as being directly related to the processes of nucleation, germination and growth of aluminum grains [51]. One of the theories involves both homogeneous and heterogeneous nucleation. Thus, during the solidification of a pure metal, the critical survival size of a nucleating particle is given by the following formula [3,52,53]:

(12) $$r*{\;}_{\mathrm{homogeneous}}=\frac{-2\gamma_{sL}}{\Delta G_{\nu }}$$

With respect to the free energy barrier, ΔG*

(13) $$\Delta G*{{\;}_{\mathrm{homogeneous}}}_{\;}=\frac{16\pi \gamma_{sL}^{3}}{3\Delta G_{\nu }^{2}}$$

where *γsL* is the surface energy at the solid–liquid interface (J/m^2^). If we assume that the specific heat of the liquid and the solid are similar, Δ*Gυ* becomes the driving force of so-lidification, namely, that

(14) $$\Delta G_{\nu }=\Delta T\Delta S=\frac{\Delta H_{f}\Delta T}{T_{m}}$$

Here, Δ*T* represents the undercooling under the liquidus (°C), Δ*S* is the change in entropy following the transition from liquid to solid state (J/°C/m^3^), Δ*H_f_* is the enthalpy of solidification, and *T_m_* is the melting temperature. If a solid seed is larger than the critical radius, r*_homogeneous_, it will survive and become a particle for nucleation. For heterogeneous nucleation, the critical size of a particle for nucleation is given by:

(15) $$r*{\;}_{\mathrm{heterogeneous}}=\frac{-2\gamma_{sL}}{\Delta G_{v}}$$

Equations (12) and (15) are identical for both types of nucleation (homogeneous and heterogeneous). The free energy barrier is given by [3,52,53]:

(16) $$\Delta G*{{\;}_{\mathrm{heterogeneous}}}^{\;}=\frac{16\pi \gamma_{sL}}{3\Delta G_{\upsilon }^{2}}f(\theta )$$

where *f*(*θ*) is a function of the contact angle *θ* on the substrate on which nucleation occurs. Thus, if *f*(*θ*) is always ≤ 1, the critical free energy for heterogeneous nucleation is therefore always less than or equal to that for homogeneous nucleation [3,52,53]. However, the most efficient heterogeneous nucleating particles are those whose angle *θ* is minimal (as close to zero as possible). Moreover, in the presence of heterogeneous nucleation, the following simplified equation applies for a heterogeneous nucleation rate per unit volume (m^−3^s^−1^):

(17) $${{{I_{\upsilon }}^{\;}}_{\mathrm{heterogeneous}}}^{\;}=10^{18}N_{\nu }^{p}\exp\left[\frac{-16\pi \gamma_{sL}^{3}f(\theta )}{3K_{B}\Delta S^{2}\Delta T^{2}}\right]$$

where *K_B_* is the Boltzmann’s constant (J/K), Nυp is the number of nucleation sites per unit volume (m^−3^), and Iυ_heterogeneous_ is the heterogeneous nucleation rate (nucleation number/m^3^s). Therefore, if the contact angle is minimal (close to zero), the wettability of the substrate is better, which improves nucleation [52,53] and, thus, the rate of nucleation increases.

**Figure 11:**
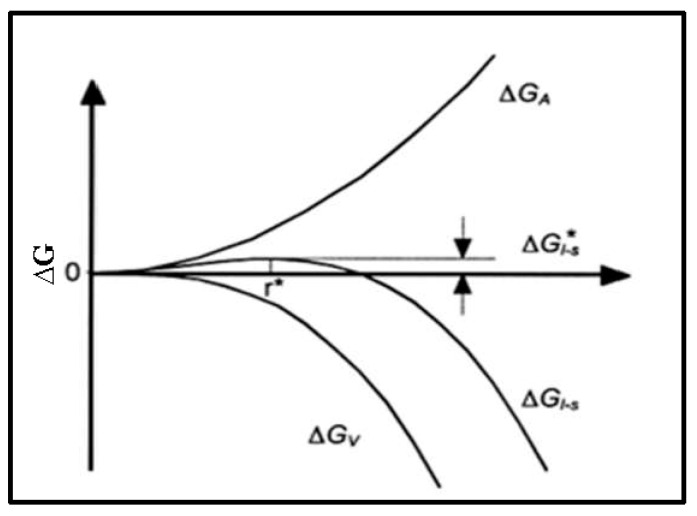
Evolution of the free energy associated with the formation of a germ of size r.

Figure 11 clearly illustrates the evolution of the free energy as a function of the radius of the seed. It is clear that the critical radius r* and the total free energy ∆G* are inversely proportional to the undercooling ∆*T*. A germ whose radius is greater than the critical radius r* tends to grow, since an increase in its radius leads to a decrease in the free energy of the system. On the other hand, if r < r*, the germ will tend to dissolve in the liquid.

**Figure 12:**
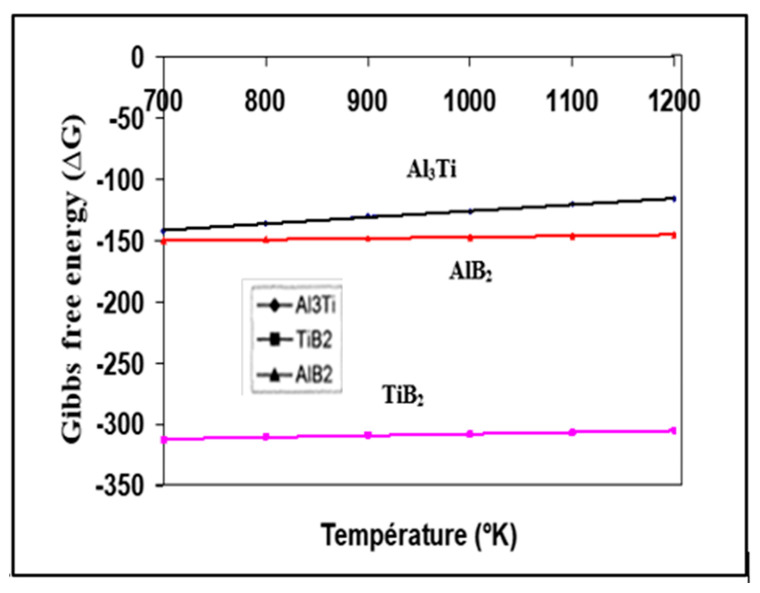
Gibbs free energies of TiB_2_, AlB_2_, and Al_3_Ti as a function of temperature (data from [54]).

When the nucleation sites are homogeneously dispersed in the liquid pool, a fine grain structure would be obtained. The important topics for understanding the nucleation phenomena may be summarized as follows: the contact angle between the molten metal and the nucleation particles; the interface energy between the molten metal and the nucleants; and the coherence of the lattices of the nucleants and liquid metal. The presence of possible phases at different T in the liquid pool can be evaluated by comparing the free energy ∆G of the reactions. Based on thermodynamic data, the calculated results are shown in Figure 12. It can be observed that ∆G for TiB_2_ is much more negative than ∆G for Al_3_Ti and ∆G for AlB_2_ in the range of T from 700 to 1200 °K, while ∆G for Al_3_Ti is less negative than ∆G for AlB_2_. In other words, the TiB_2_ phase is easier to form than the Al_3_Ti and AlB_2_ phases. With increasing temperature, the changes of free Gibbs enthalpy of TiB_2_ and AlB_2_ are almost constant, while that of Al_3_Ti becomes small. In fact, the theoretical prediction indicates that Al_3_Ti particles become unstable when the reaction temperature is increased. From the crystallographic point of view, and to further explain the high stability of the TiB_2_ nucleation sites, the hybridization of the 3d orbital of Ti and the 2p orbital of B is the main reason for the strong bond between these two elements. The nature of the bonding behavior between Ti and B layers is a combination of covalent and ionic types [54].

## 8. Solidification Parameters

**Figure 13:**
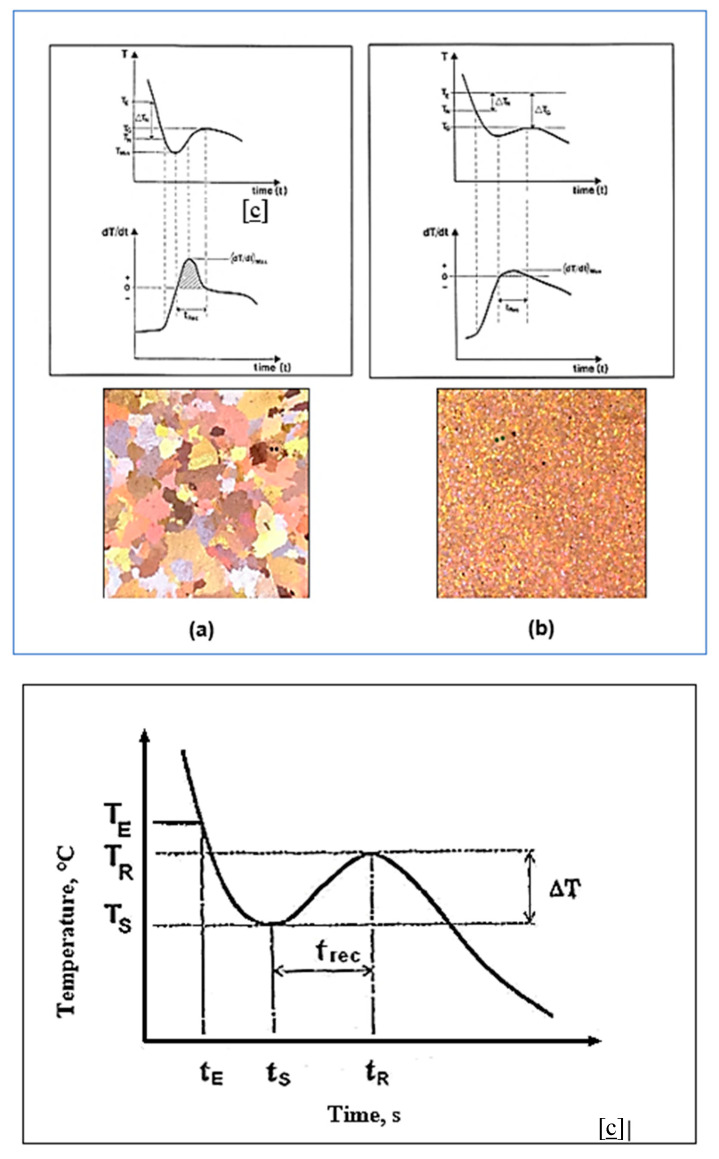
(**a**) First part of a cooling curve and its derivative obtained from liquid metal close to the wall of the casting mold. (**b**) Cooling curve and its derivative of a sample to which titanium boride particles have been added. The nucleation temperature is below the liquid metal growth temperature. The recalescence phenomenon shows a very low value of (*dT*/*dt*)_max_, indicating a sample whose grains are refined. Other temperature parameters are found in data ([55]). (**c**) Effect of different parameters on the start of solidification of α-Al dendrites.

Grain refinement is a result of two separate processes: nucleation of new liquid aluminum crystals, followed by growth to a limited size. Both of these processes need a driving force which must be provided to the system through undercooling and supersaturation with respect to the equilibrium conditions of the real system. Figure 13a shows that during the first period of the solidification process, some parts of the liquid metal which are in direct contact with the inner walls of the mold are cooled to allow for the nucleation of new aluminum grains. Figure 13b depicts the start of nucleation above the steady state growth temperature. When an alloy solidifies, different phases are formed. Energy (latent heat) is released. This release of energy counterbalances the energy that is lost by the metal as it cools. This results in a local and temporary decrease in the rate of cooling. Thermal analysis, which is used to examine this phenomenon (by studying the plots of temperature versus time), makes it possible to study solidification and to characterize the macrostructure and the microstructure to a certain extent [55].

The symbols refer to the following:

T_E_ = The liquidus equilibrium temperature.
T_G_ = The steady state growth temperature of the molten metal.
T_N_ = The onset of nucleation temperature.
T_MIN_ = The temperature at which the newly nucleated crystals have grown to such an extent that the latent heat released swings out of equilibrium. The period of time required for this reaction is termed the recalescence period (t_Rec_).

The examining process begins with the analysis of the solidification curve. First, the cooling of a solid body without solidification or phase change does not present any particularity due to the absence of a physico-chemical reaction. The only observation is the gradual decrease in the cooling rate due to the decrease in the temperature gradient between the solid and its environment. During the solidification of a pure metal, the presence of a plateau is observed. Solidification of a solid solution alloy shows no plateau. Eutectic alloys will solidify as pure metal. Finally, the graph of the solidification of hypoeutectic or hypereutectic alloys will combine the features of a solid solution alloy and that of an eutectic alloy [6,54,56].

The solidification of any alloy or metal is accompanied by some particular phenomena [57,58]. First, since the solidification rate is not infinitesimal (i.e., perfectly balanced), undercooling (∆T_S_) or “undercooling” [34,55,59,60,61,62] is possible (Figure 13). Solidification does not begin at the equilibrium temperature (T_E_) but at the undercooling temperature (T_S_). Thus, the undercooling, ∆T, is calculated from the following equation:

(∆T) = T_E_ − T_S_ = (K_1_ + K_2_) Ṫ (18)

where ∆T_S_: undercooling (°C); T_E_: T equilibrium (°C);

T_S_: T undercooling(°C); K_1_: constant (s);

K_2_: constant (s); Ṫ: rate of solidification (°C/s).

The maximum temperature is called the “recalescence temperature” (T_R_). The latent heat emitted during the onset of solidification, when the alloy reaches the temperature T_S_, partly explains this phenomenon; see Figure 13c. It is sometimes possible to establish a correlation between grain size and ∆T or t_rec_ [57,63,64]. Moreover, ∆T and t_rec_ can sometimes be combined under a single variable, the Liquidus Peak Parameter (PPL). To obtain this parameter, the first derivative [65] (*dT*/*dt*) of the solidification curve is first calculated, then the area under this curve (the hatched surface) is evaluated. As with ∆T and t_rec_, there is sometimes a correlation between grain size and this parameter [63,64]. During the solidification of this alloy, two main phases can be identified. First, at 610 °C, the formation of the dendritic network of α-Al begins. Then, the second main phase, the Al-Si eutectic, appears when the temperature reaches 577 °C.

**Figure 14:**
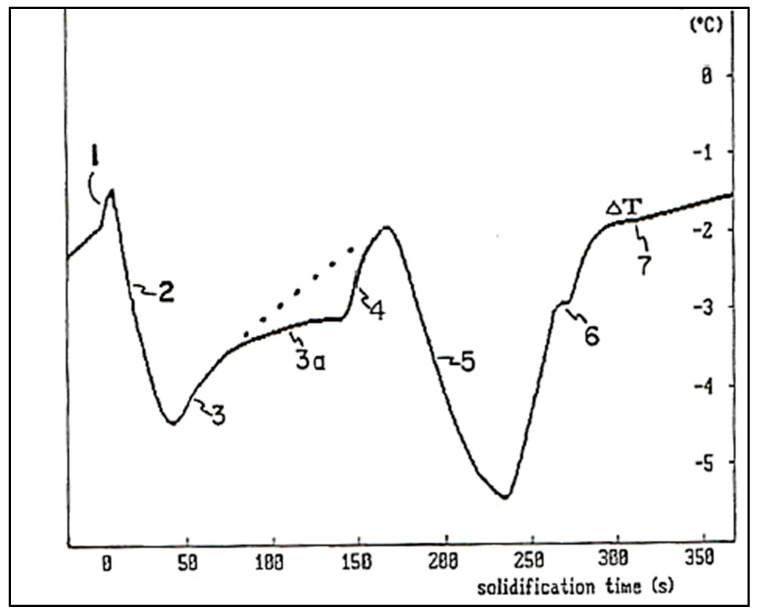
Plot of difference between T_P_ and T_C_ as a function of time (data from [55]).

It is possible to further characterize the solidification process with the use of the first derivative (*dT*/*dt* or *Ṫ*) of the solidification curve (T_(t)_) [64]. The first derivative corresponds to the slope of the solidification curve, as shown in Figure 14. It also corresponds to the cooling rate of the alloy. The formation of a new phase releases energy (latent heat), which increases the first derivative (i.e., the cooling rate decreases). In the initial part of the curve, the alloy is completely liquid and the cooling rate is about 0.9 *°C/s*. In the absence of solidification, the rate of cooling would gradually decrease over time. This dotted line is called the “zero curve derivative”. Since the cooling results from radiation as well as natural convection between the ambient air and the mold, the rate of solidification is directly related to the temperature difference between these media. It will therefore gradually decrease over time.

At point 1 of the plot (Figure 14), there is a very clear deviation of the derivative from the dotted curve. The derivative increases to reach a maximum of 0.4 °C/*s* and then decreases somewhat. In other words, when the derivative is maximum, the sample heats up at a rate of 0.4 °C/*s*. This release of energy results from the sudden nucleation of the aluminum grains (α-Al). Over time, the nucleation rate decreases and the frontal growth of the α-aluminum dendrites progresses, from the slightly cooler walls, towards the center of the mold (point 2). After some time, the dendritic network of aluminum occupies the entire volume of the metal. Any further dendrite growth will occur laterally only. This thickening of the dendrites corresponds to region 3. Note that the curve remains above the dotted curve (the basic cooling curve), due to the continual growth of crystals (grains) of aluminum (α- Al). At point 4, another rapid increase in the derivative appears which results from the sudden appearance, by nucleation, of silicon crystals (the eutectic phase). The growth of silicon (and also of solid aluminum which constitutes the eutectic) continues in region 5 of the curve. Regions 4 and 5 show that, similar to the formation of aluminum grains, the solidification of the eutectic is initially rapid, then slows down as solidification progresses. Finally, at point 6, the Mg_2_Si phase appears. Hence the importance of using the first derivative (*dT*/*dt*).

When solidification is complete, the derivative of the solidification curve returns to the base curve (or derivative of the zero curve, dotted). However, a small dip is observable just below the base line (region 7). This phenomenon is explained by the position of the thermocouple located in the center of the cylindrical mold. At the end of solidification, the energy released by the formation of the different phases suddenly ceases. There then occurs a rapid standardization of the temperature gradient of the sample from the center towards the wall, which temporarily rebalances the temperature and explains the dip in the curve at this point (region 7) [64].

Another important development in thermal analysis is the use of two thermocouples, one placed at the wall of the mold and the other at the center. This is called differential thermal analysis. This method requires that the mold be preheated before casting, which avoids any rapid cooling on the surface. Thus, the entire contents of the crucible cool evenly before solidification begins. Therefore, it is possible to detect variations in the solidification curve that result from the formation of new phases. In other words, a new phase first forms on the surface. Then solidification (and the latent heat produced) progresses towards the center of the sample.

A first observation shows that the metal close to the wall is colder than that in the center. If we calculate the difference between the temperature at the wall (T_P_) and that at the center (T_C_), i.e., T_P_-T_C_, we obtain a new curve. Since the thermal conductivity of solid aluminum is equal to that in the liquid state, the thermal gradient before and after solidification is identical (2 °C). When the solidification of the dendritic network of α-Al begins (region 1), the surface heats up and the difference between T_P_ and T_C_ decreases. As solidification progresses, more energy is generated at the center than at the surface. The difference between T_P_ and T_C_ increases. After some time, the α-aluminum dendrites occupy the entire volume of the alloy (between regions 2 and 3). This means that, at this time, all the grains are formed. Then there is only thickening of the dendrites or lateral growth (region 3). Finally, reactions 4 to 7 are exactly the same as those described previously.

## 9. Calculation of Solid Fraction

During solidification, there occurs a phase change. The proportion of solid metal gradually increases. It is possible to calculate the evolution of the solid fraction using thermal analysis. First, the cooling is assumed to be Newtonian (no internal thermal gradient). We can then propose the following equation for the mould–metal system [65]:

(energy generated by a phase transformation) − (energy lost by the metal) = (energy transmitted to the mold)

(19) $$\frac{dQ_{L}}{dt}-V•\rho •C_{p}\frac{dT}{dt}=hA(T-T_{0})$$

where:

- *Q_L_*: latent heat of solidification (J);
- *V*: volume of the sample (m^3^);
- *ρ*: volumetric mass (kg/m^3^);
- *Cp*: specific heat of metal (J/kg °C);
- *T*: temperature of the metal (°C);
- *t*: time (s);
- *h*: coefficient of heat transfer (J/m^2^s °C);
- *A*: surface (m^2^);
- *T*_0_: ambient temperature (°C).

After reorganization, the above equation can be expressed as:

(20) $$\frac{dT}{dt}=\frac{1}{V\rho C_{p}}\left[\frac{dQ_{L}}{dt}-hA(T-T_{0})\right]$$

The last equation is associated with the solidification curve [66,67]. In the absence of the formation of a phase, Equation (20) reduces to:

(21) $$\frac{dT}{dt}=-\frac{hA(T-T_{0})}{V\rho C_{p}}$$

This last equation is called the “zero curve derivative”. This curve can then be plotted, as well as the solidification curve and its first derivative. Combining Equations (20) and (21) gives:

(22) $$\frac{dQ_{L}}{dt}=V\rho C_{p}\left[{\left(\frac{dT}{dt}\right)}_{cs}-{\left(\frac{dT}{dt}\right)}_{cz}\right]$$

The subscripts “cs” and “cz” represent the solidification curve and the zero curve, respectively. After integration, Equation (22) becomes:

(23) $$Q_{L}=V\rho C_{p}\int_{0}^{t_{s}}\left[{\left(\frac{dT}{dt}\right)}_{cs}-{\left(\frac{dT}{dt}\right)}_{zc}\right]dt$$

Or, 

L = Q_L_/Vρ (24)

L = Cp (Area under the derivative of the solidification curve − the area under the derivative of the zero curve)

**Figure 15:**
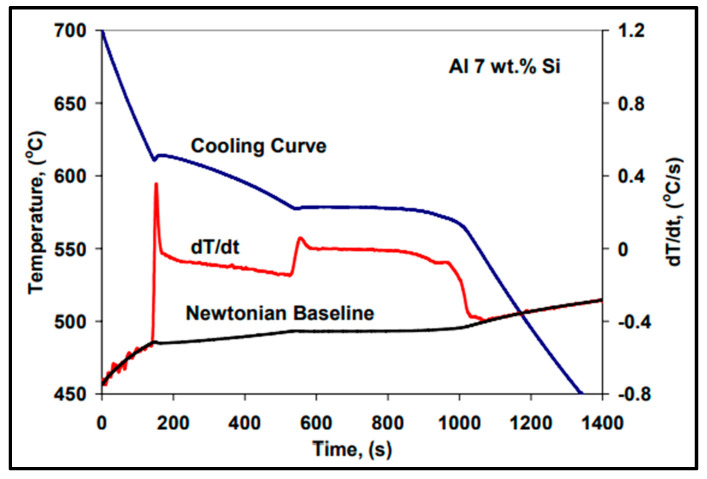
Calculation of the solid fraction as a function of solidification time (data from [69]).

The variable “L” indicates the latent heat of solidification, and ts is the solidification time. From Equation (24), the latent heat of solidification can be integrated numerically. Finally, the solid fraction fs at time t can be calculated by evaluating the ratio between the surface covered at time t and the total surface [66,67,68] (Figure 15)

(25) $$f_{s}=\frac{\int_{0}^{t}\left[{\left(\frac{dT}{dt}\right)}_{cs}-{\left(\frac{dT}{dt}\right)}_{cz}\right]dt}{\int_{0}^{s}\left[{\left(\frac{dT}{dt}\right)}_{cs}-{\left(\frac{dT}{dt}\right)}_{cz}\right]dt}\;f_{s}=\frac{C_{p}}{L}\int_{0}^{t}\left[{\left(\frac{dT}{dt}\right)}_{cs}-{\left(\frac{dT}{dt}\right)}_{cz}\right]dt$$

With Equation (25), it is possible to calculate fs by numerical integration. In addition, it allows for the obtaining of different plots. Moreover, it is possible to calculate the rate and amount of energy released during the formation of the different phases [68,69].

The dendrite coherence point appears in the A356.2 alloy at a solid fraction of 23%. This means that a sample of this alloy is completely filled with aluminum dendrites after one-quarter of the latent heat of solidification has been released. This characteristic of the alloy has an influence on its castability and its fluidity. In fact, when this point is reached, the macrostructure and, in particular, the grains are almost definitively fixed. Moreover, the sample presents a certain degree of rigidity which results from the embedding of the dendritic network. Therefore, from this point, some problems such as sink marks and porosity begin to appear. Furthermore, it is also from this point that stresses can be initiated and lead to hot cracking [70].

## 10. Duplex Theory of Nucleation

The duplex theory was proposed by Guthrie [71], although more persuasive evidence was provided by Schumacher and Greer [72,73]. Mohanty et al. [74,75] added synthetic TiB_2_-like particles of about 5 µm in diameter to a liquid aluminum metal, with various concentrations of dissolved titanium. When there was no excess titanium, no grain refinement was observed, which means that TiB_2_ is a poor nucleant; moreover, TiB_2_ particles were observed at grain boundaries. However, when titanium was added in excess, a significant improvement in grain refinement was observed and borides were found at the grain centers. At hyperperitectic titanium concentrations, an Al_3_Ti layer was formed on the surface of the TiB_2_ particles, around which was an α-Al layer. But even at the hypoperitectic additions, it appeared that layers between TiB_2_ and α-Al had been formed. Their evidence for what occurs at hypoperitectic concentrations is based on an extrapolation of observations at hyperperitectic concentrations and on the observation of what may be a layer formed on a TiB_2_ particle, which appeared to have an increased titanium concentration [76].

**Figure 16:**
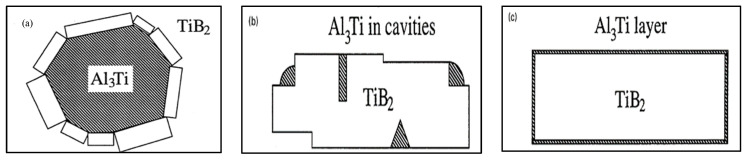
(**a**) Surrounding layer of TiB_2_; (**b**) precipitation of Al_3_Ti on edges and/or in holes; (**c**) accumulated layer of Al_3_Ti (data from [72]).

Concurrently, Schumacher and Greer [72,73] also performed experiments on embedding a conventional aluminum grain-refiner-based Al-Ti-B alloy system in a glassy matrix. The rod contained Al_3_Ti and TiB_2_ particles (nucleant particles). During crystallization from the glassy state, nucleation and growth of α-Al could be observed on TiB_2_ particles coated with a layer of Al_3_Ti. Empirical relations found in casting practice of Al-alloys, such as excess Ti necessary for grain refinement, can be related to the observed nucleation mechanism, which is found to be very sensitive to both crystallographic and chemical factors, Al-Ti-B, in that borides were covered by a layer of Al_3_Ti, which was also covered by α-Al. Therefore, they proposed the same nucleation order as Mohanty et al. [74,77]. The orientation ratios between the phases have also been characterized and are presented in Table 3 below. The authors also found that, after annealing, the Al_3_Ti layer sometimes remained the same size but at other times grew, while the α-Al crystals grew significantly in all cases. The data in Table 3 were obtained using a TEM with the electron beam aligned parallel to the <1120> orientation of the boride. Figure 16 depicts the proposed schematic of grain nucleation when Ti or B is added to the molten metal.

One of the most interesting suggestions in the work of Schumacher and Greer [72,73] is that the aluminide layer forms in liquid aluminum at temperatures of 1300 °C and that it grows sustaining this temperature. The results and conclusions of Schumacher and Greer [65,66] have been supported by Kearns et al. [7]. AlB_2_ has proven to be a good nucleant in foundry alloys without the presence of titanium. Experiments using TiB_2_ or AlB_2_ without excess titanium should be performed to determine if they are able to nucleate α-Al without an Al_3_Ti layer being present.

Mohanty et al. [78] suggest that the formation of Al_3_Ti is caused by a titanium concentration gradient towards the boride particles, constituted by an activity gradient towards the borides. Due to the local equilibrium near the borides, the Al_3_Ti would be stable and could subsequently nucleate in the α-Al phase, as is the case for alloys whose titanium is found in hyperperitectic concentrations. Bäckerud et al. [18] supported this titanium gradient theory of segregation. Naess and Berg [79] suggested the existence of a fairly high concentration of titanium surrounding the borides in the liquid zone.

**Figure 17:**
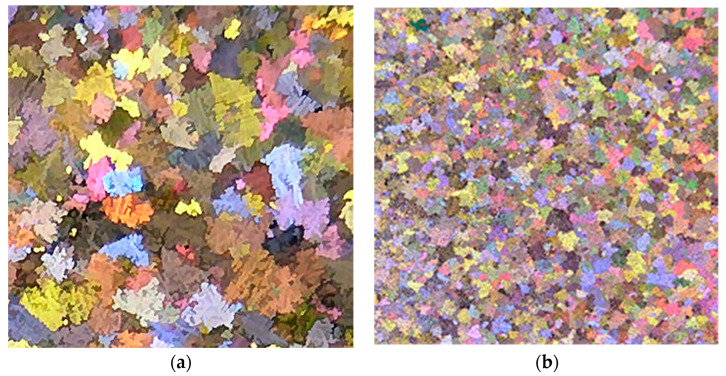

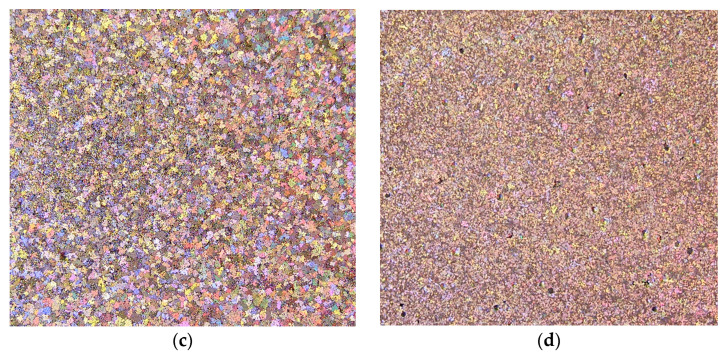
Evolution of the grain size of the A356 alloy: (**a**) no addition, (**b**) Al-10%Ti, (**c**) Al-5%Ti-1%B, (**d**) Al-4%B. All micrographs are at the same magnification.

The duplex theory of nucleation is not totally new. In 1971, Bäckerud [18] proposed a series of reactions to explain this theory. Cornish [80] suggested that the role of borides is to enhance the formation of Al_3_Ti at hypoperitectic concentrations, based on the claim of Mohanty et al. [74] about the segregation of titanium to borides. If the TiB_2_ particles nucleate the Al_3_Ti particles, these, in turn, nucleate the α-Al. If Al_3_Ti particles form on the surface of TiB_2_ particles that increase nucleation, then it is the borides that act directly or indirectly as nucleation sites. Figure 17 shows the effect of the applied grain refiner on the alloy grain size. In the case of a non-treated alloy, the grain size is approximately 1850 µm in the base alloy; see Figure 17a. After an addition of 0.08% Ti using the Al-10%Ti master alloy, the size drops to around 800 µm; see Figure 17b. The aluminum grain size continues to decrease up to less than 0.1% Ti, added in the form of the Al-5%Ti-1%B master alloy; see Figure 17c. A minimum grain size is achieved using Al-4%B, reaching about 200 µm; see Figure 17d. Adding excess titanium or boron has no effect on reducing the aluminum grain size. On the contrary, an overdose of Ti or B can result in a deleterious impact on the alloy microstructure and properties.

## 11. Combined Effect of Ti/B and Sr

**Figure 18:**
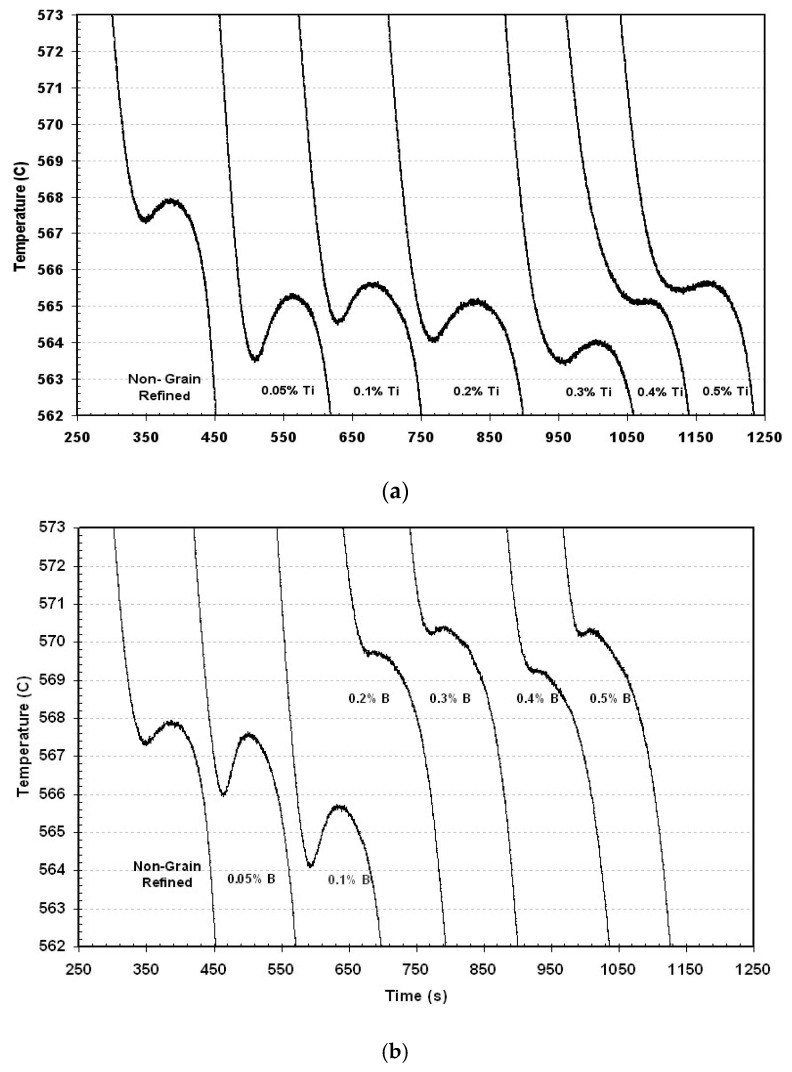
(**a**) Eutectic temperature in the A356 alloy after addition of Sr and gradual addition of Ti at various holding times (30, 60, 90 min). (**b**) Solidification curves for thermal analysis of 200 ppm Sr-modified A356.2 alloy with different B additions as Al-4%B.

**Figure 19:**
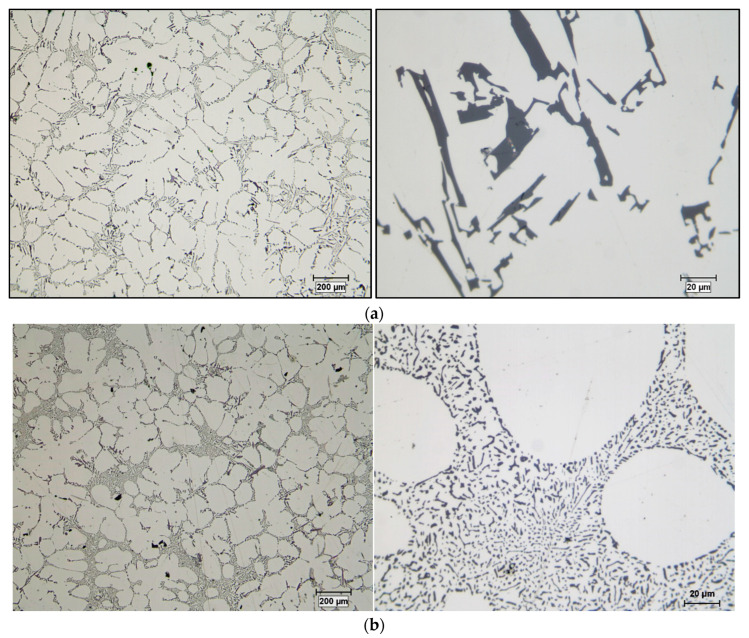
Optical micrographs showing the combined effect of modifier and refiner on the shape of α-Al dendrites and eutectic Si particles in A356 alloy: (**a**) without addition, (**b**) with 0.1% Ti, 200 ppm Sr addition.

The thermal analysis curves presented in Figure 18a (obtained from the 356 alloy) clearly show that the gradual addition of titanium has no effect on the nucleation and growth temperatures of the eutectic Si. Although the Ti content increases in the presence of strontium, the combined effect on the thermal characteristics of Si is the same as that of the addition of strontium alone. To demonstrate the affinity existing between the two agents, a specimen was cast after a period of two hours to give more chance to a predicted reaction between titanium and strontium. This proves the absence of interaction that can exist between these two elements and that there is no “poisoning” effect on the finesse of the Si eutectic phase. Therefore, grain refinement and strontium modification occur almost entirely, as can be seen in Figure 19.

**Figure 20:**
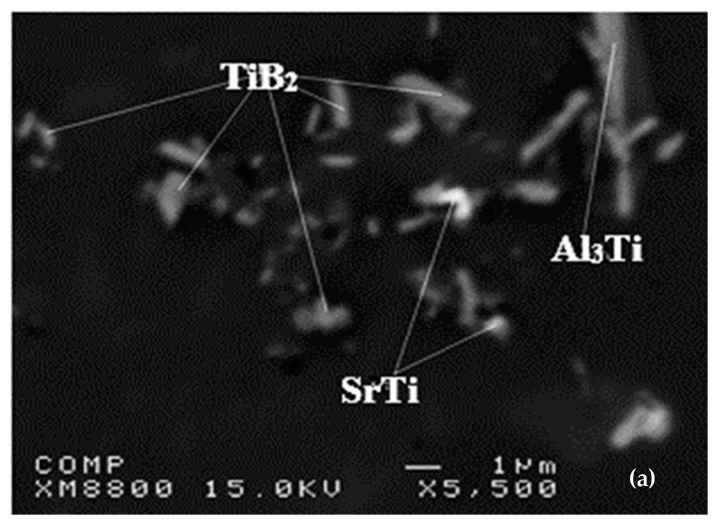

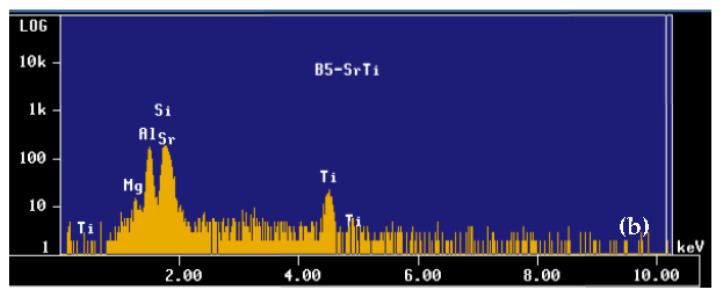
(**a**) Backscattered electron image of different particles in the sample grain refined with the Al-5%Ti-1%B master alloy as 0.5% Ti, (**b**) EDS analysis of the particles, with SrTi as pointed out in (**a**).

Figure 18b shows a corresponding set of solidification curves for additions of boron ranging from 0.05–0.5%. This figure illustrates two distinct regions: one in which the eutectic temperature decreases when the boron addition is less than approximately 0.20 %B; and the other in which the eutectic temperature increases rapidly, rising even beyond the original temperature when B addition increases from approximately 0.20 to 0.50%B. It will be observed, therefore, that when the B increases, the eutectic growth temperature drops initially by almost 2 °C until 0.1%B is attained, but when the boron is increased to higher levels, the eutectic growth tem¬perature also increases. This behavior indicates that the interaction of Sr with B affects the modification of Si in A356.2 alloys, on condition that the amount of boron is raised by more than 0.1%.These results are consistent with those obtained by Asensio et al. [81]. From the map showing the distribution of the elements in the precipitated phases, Sr is found in regions far from those where the titanium is found, as illustrated by Figure 20.

A number of researchers have found the interaction of Al-Sr modification and Al-Ti-B refinement. Liao and Sun [82] reported poisoning at high levels of Sr and B. Faraji and Katgerman [83] also reported the poisoning effect of Sr and Ti. Suárez-Peña and Asensio-Lozano [84] found that, at a higher Ti/Sr ratio (Ti:Sr = 1:1), the poisoning effect is more pronounced than at a lower Ti/Sr ratio (Ti:Sr = 3:5). However, Ibrahim et al. [85] stated that combined addition of grain refiners along with Sr to the A356 alloy could result in reducing the grain size of α-Al dendrites as well as changing the morphology of the eutectic Si particles.

Figure 20 displays the backscattered (BS) image and the corresponding EDS analysis of the particles observed in the image of the A356.2 alloy, containing 0.52% Ti and 220 ppm Sr in the as-cast condition. The sample exhibits a large number of fine particles (1 μm or less in diameter), which were believed to be Sr-Ti compounds. To determine their chemical composition, an EDS analysis was conducted at 15 kV, but since the Kα line of the silicon element is very close to the Lα line of the Sr element, any interaction between Ti and Sr cannot be concluded with certainty. Such particles appeared in the corresponding images of the samples refined with Al-2.5Ti-2.5B and Al-1.7Ti-1.4B as well [10,86].

## 12. Grain Size–Mechanical Properties Relationship

In general, reducing the grain size improves the mechanical properties. For some metals and alloys, a mathematical expression links the grain size with the yield strength (Re_0.2_). This is the Hall–Petch [80] equation:

Re_0.2_ = σ_0_ + k∗d ^−1/2^ (26)

where:

Re0.2: conventional elastic limit (MPa);
σ_0_: constant (MPa);
k: parameter whose value depends on the metal;
d: grain size (mm or µm).

**Figure 21:**
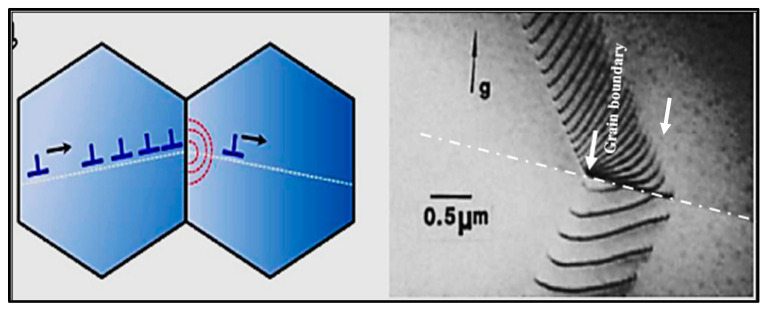
Dislocation pile-up during plastic deformation (data from [82]).

However, unlike metals with a c.c. structure (centered cubic lattice), the value of k is small for metals with a c.f.c. structure. (face-centered cubic lattice), like aluminum, and h.c. (hexagonal compact system). Consequently, certain mechanical properties (Re_0.2_, etc.) vary little as a function of grain size [82]; see Figure 21.

Nevertheless, the reduction in the size of the grains makes it possible to increase the number of microstructural barriers to the propagation of cracks [82]. Moreover, the formation of smaller grains has direct consequences on the various mechanisms of the formation and propagation of cracks [83]. This results in improved fatigue properties and toughness. Moreover, the reduction in the grain size improves the distribution of the porosity, which also attributes to an increase in the toughness [84]. It should be noted that the modification of the Al-Si eutectic has little effect on the toughness [85,86]. Obviously, fatigue properties are optimal when porosity and surface defects are minimal [10].

**Figure 22:**
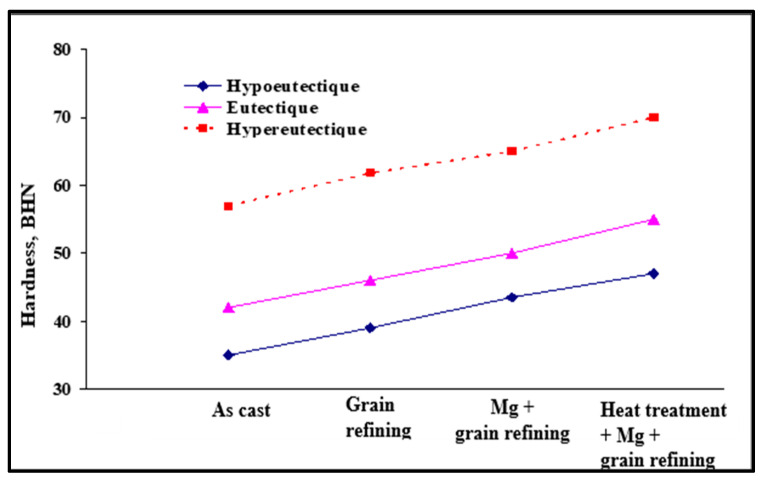
Effect of various treatments on the hardness of Al-Si cast alloys (data from [91]).

**Figure 23:**
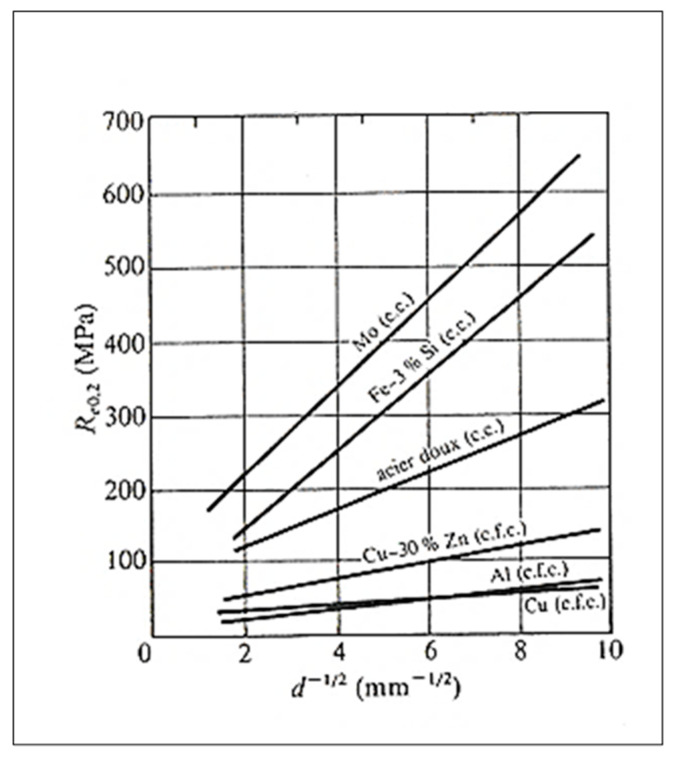
R_e0.2_ as a function of alloy composition (data from [93]).

The improvement of the mechanical properties following the increase in the rate of solidification does not only result from the reduction in the size of the grains; this improvement is also explained by the decrease in the interdendritic space (DAS or “dendrite arm spacing”) [87]. Furthermore, the increase in the rate of solidification minimizes the size of the porosities and consequently improves the mechanical properties [88]; see Figure 22 and Figure 23. The combined treatments of grain refining and modification (of the Al-Si eutectic phase) make it possible to enhance the alloy tensile strength as well as the ductility [89,90,91,92,93].

## 13. Conclusions

Based on the information documented in the present review, the following conclusions may be drawn:

1- Undercooling and recalescence temperatures increase with the initial increase in titanium concentration. If the concentration reaches approximately 0.25%, a rapid decrease in these temperatures is observed. Thereafter, the temperatures increase again with Ti concentration and eventually become constant.
2- The undercooling parameter decreases as a function of the Ti concentration and, from a concentration of around 0.20%, this parameter becomes zero.
3- For the recalescence temperature parameter, for a concentration of more than 0.20% Ti, it can be zero. Otherwise, the number of experimental results is insufficient to determine the exact variation.
4- The presence of excess silicon in Al-Si alloys leads to a strong interaction between titanium and silicon. This high affinity leads to the formation of (Al,Si)_3_Ti-type phases, weakening the nucleation opportunities of the dendritic phase and consequently reducing the degree of grain refinement. The titanium disilicate phase tends to form more when the liquid metal is held for long periods.
5- For master alloys, residual titanium Ti in alloy A356 reacts with boron B to form TiB_2_, which subsequently acts as an active seed alongside AlB_2_ for the α-Al phase.
6- A combined treatment (refining and modification) is more advantageous for the grain size and the shape of the eutectic Si than when the treatments are carried out individually. This is quite evident in the case of the addition of strontium and titanium in the form of master alloys to the A356 alloy.
7- The introduction of AlB_2_ in the form of Al-4%B in alloys containing traces of titanium leads to the reaction between boron and titanium to form TiB_2_. Grain refining is achieved primarily with TiB_2_ rather than AlB_2_, or both, depending on the Ti content in the given alloy.
8- When strontium is added, the boron reacts with the strontium to form compounds of the SrB_6_ type, which is supposed to be a very weak refiner. The affinity between titanium and boron is higher than the affinity existing between boron and strontium.

## Figures and Tables

**Table 1:** Chemical composition of the A356.2 alloy.

Alloy	Al	Si	Cu	Mg	Fe	Mn	Zn	Ti	Sr
A356.2	bal.	6.78	0.02	0.33	0.11	0.04	0.04	0.07	0

**Table 2:** Chemical composition (at. %) of the platelets in Figure 9d.

Spot #	Al	Ti	Si
1	71.340	23.412	2.68
2	71.445	23.497	2.70
3	71.576	23.019	2.09
4	71.847	23.653	1.66

**Table 3:** Crystallographic orientations between TiB_2_, Al_3_Ti, and α-Al.

Number	TiB_2_	Al_3_Ti	α-Al
1	<1120> {0001}	<110> {112}	<110> {111}
2	<1120> {0001}	<210> {112}	<110> {111}

## Data Availability

Data will be made available upon request.
